# Visual cortex recruitment during language processing in blind individuals is explained by Hebbian learning

**DOI:** 10.1038/s41598-019-39864-1

**Published:** 2019-03-05

**Authors:** Rosario Tomasello, Thomas Wennekers, Max Garagnani, Friedemann Pulvermüller

**Affiliations:** 1 Brain Language Laboratory, Department of Philosophy and Humanities, WE4 Freie Universität Berlin, Habelschwerdter Allee 45, 14195 Berlin, Germany; 2 Berlin School of Mind and Brain, Humboldt Universität zu Berlin, Luisenstraße 56, 10117 Berlin, Germany; 3 Centre for Robotics and Neural Systems (CRNS), University of Plymouth, A311 Portland Square Building, PL4 8AA Plymouth, Devon United Kingdom; 4 Department of Computing, Goldsmiths, University of London, SE14 6NW London, United Kingdom; 5 Einstein Center for Neurosciences, Charitéplatz 1, 10117 Berlin, Germany

## Abstract

In blind people, the visual cortex takes on higher cognitive functions, including language. Why this functional reorganisation mechanistically emerges at the neuronal circuit level is still unclear. Here, we use a biologically constrained network model implementing features of anatomical structure, neurophysiological function and connectivity of fronto-temporal-occipital areas to simulate word-meaning acquisition in visually deprived and undeprived brains. We observed that, only under visual deprivation, distributed word-related neural circuits ‘grew into’ the deprived visual areas, which therefore adopted a linguistic-semantic role. Three factors are crucial for explaining this deprivation-related growth: changes in the network’s activity balance brought about by the absence of uncorrelated sensory input, the connectivity structure of the network, and Hebbian correlation learning. In addition, the blind model revealed long-lasting spiking neural activity compared to the sighted model during word recognition, which is a neural correlate of enhanced verbal working memory. The present neurocomputational model offers a neurobiological account for neural changes following sensory deprivation, thus closing the gap between cellular-level mechanisms, system-level linguistic and semantic function.

## Introduction

The classical model of the neurobiology of language, based on brain lesion data^1,2^, proposed a left-lateralized linguistic network of the fronto-temporal regions located around the perisylvian fissure^3^. However, recent neuroimaging studies, as well as patient data, reported a more detailed cortical organization of the language areas, showing that brain areas outside the classical perisylvian cortex as well contribute to the processing of meaningful symbols and language^4–6^. A range of cortical areas have been documented to be differentially involved, depending on the semantic type of symbols or larger meaningful constructions^7–14^. For example, Moseley *et al*.^14^, reported enhanced neuromagnetic (MEG) responses for action words in the fronto-central areas, including motor regions, and for object-related words in the visual temporo-occipital areas, respectively. This and similar observations support neurobiological language models postulating that linguistic and semantic processes are carried by neuron circuits distributed across the perisylvian language regions as well as modality-preferential and multimodal areas in ‘extra-sylvian’ space^6,15–18^.

**Figure 1:**
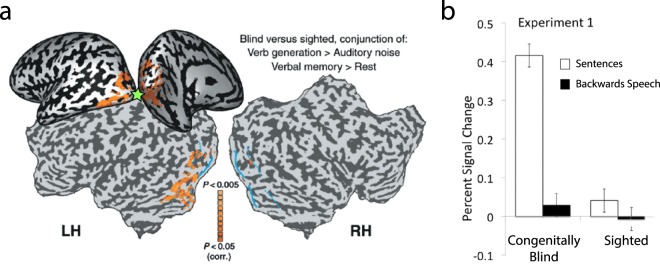
FMRI activation patterns  in congenitally blind and sighted individuals. (**a**) Activation of the primary (V1) and higher extra-striate visual areas when blind people recall words from memory or generate verbs from nouns compared to the sighted individuals (adapted from Amedi *et al*.^22^). The green asteriks indicates the stimulated cortical area delivered with rTMS causing substantial semantic errors in the verb generation task (adapted from Amedi *et al*.^25^). (**b**) Percent signal change in the left primary visual area for blind and sighted control participants during meaningful sentence comprehension and backwards speech perception (adapted from Bedny *et al*.^30^, this figure is not covered by the CC BY licence. [Credits to National Academy of Science]. All rights reserved, used with permission).

A range of studies reported that the distributed language network shows striking capabilities to re-organize and adapt to focal lesions or sensory deprivation^19–21^. Compared with healthy individuals, blind people’s language processing in the so-called verb generation task leads to relatively stronger activation of visual areas in occipital cortex^22–27^. Several brain imaging studies showed activation of the primary visual (V1) and higher extra-striate visual cortices when congenitally blind individuals were required to generate semantically related verbs to heard nouns^22–24^ (see Fig. 1a). In contrast, sighted subjects showed activation of the perisylvian language regions (e.g., Broca’s and Wernicke’s areas) and motor areas, but no or significantly less visual area activation than blind individuals^23,24^. Similar differences in V1 activation have also been reported for single word^27,28^ and sentence processing tasks^29,30^, which imply semantic understanding^27–29^. Furthermore, congenitally blind people with relatively stronger V1 activity in the processing of meaningful language were reported to show better verbal working memory^22^ and generally enhanced verbal abilities compared to sighted individuals^22,31–33^. Although one might argue that visual responses in blind individuals are epiphenomenal with no functional relevance for language processing, a study inducing temporary virtual lesions of the primary visual area (V1) using transcranial magnetic stimulation (TMS) during a verb generation task showed an increase in semantic (but not phonological) errors in blind individuals. In contrast, sighted control subjects showed a similar behavioural change only when TMS was applied to the left prefrontal cortex (lPFC)^25^. These results demonstrate that, in congenitally blind subjects, visual cortices respond in a similar way as classic language regions^30^ and are functionally relevant for language and semantic processing.

Undeprived healthy individuals may also activate their visual areas in language processing, but this is specific to words and sentences with a strong semantic relationship to visual information, for example, words like ‘cow’ or ‘tower’, which have visually perceivable referents^8,14,34–36^. Associative learning can explain this category-specific semantic activation in the human brain: Because symbols with ‘visual semantics’ frequently co-occur with visually perceived referent objects during learning^37^, the correlated neuronal activations are mapped at the neuronal level. However, such stimulus-driven correlation is obviously impossible in congenitally blind subjects. Therefore, the generally robust visual cortex activations during language processing and the associated relevance of visual areas in the blind appear as a mystery.

Why is the visual cortex generally relevant in language processing in congenitally blind individuals, and why would a role of visual areas in sighted subjects, if present at all, be restricted to only specific semantic categories?

It is unlikely that congenitally blind and undeprived human subjects differ in the neuroanatomical connections interlinking visual areas and language regions, as diffusion tensor imaging (DTI) studies do not consistently demonstrate such differences^38–41^. However, at the functional level, there is evidence for relatively stronger functional connectivity (estimated from fMRI) between visual and frontoparietal language regions in blind people^30,42–44^. Therefore, the critical question to answer is how, given the absence of differences in anatomical long-range connectivity, it is possible that visual cortex function changes in congenitally blind people. It has been suggested that the lack of competing inputs to the deprived cortical areas during development may be critical; this would leave the blind’s visual cortices available for recruitment for language processing^45^. However, the neural mechanisms determining such takeover remain to be specified. Here, we show that general neurobiological mechanisms and principles can explain the functional changes in the visual cortex, and we identify the factors that may drive such plastic change.

We applied a neurobiologically constrained model implementing properties of fronto-temporo-occipital areas and their connectivity in an attempt to simulate features of language acquisition in undeprived (i.e. sighted) and deprived (i.e. congenitally blind) human subjects. The models were given information for learning the referential relationships between individual verbal symbols and the actions and objects they are typically used to communicate about. By comparing (congenitally) ‘blind’ and ‘undeprived’ models, we aimed to shed light on the neural language mechanisms consequent to sensory deprivation.

## Results

### General model architecture

**Figure 2:**
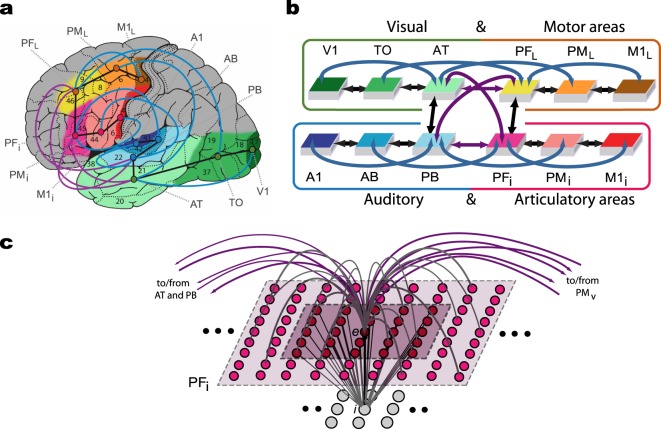
Model of lexical and semantic mechanisms. (**a**) Structure and connectivity of 12 frontal, temporal and occipital cortical areas relevant for learning the meaning of words related to actions. Perisylvian cortex comprises an inferior-frontal articulatory (red colours) and a superior temporal auditory (blue colours) system, and the extrasylvian areas comprise a lateral dorsal hand-motor system (yellow to brown) and a visual ‘what’ stream of object processing (green). Numbers indicate Brodmann Areas (BAs) and the arrows (black, purple and blue) represent long distance cortico-cortical connections as documented by neuroanatomical studies. (**b**) Schematic global area structure and connectivity of the implemented model. The colours indicate correspondence between cortical and model areas (panels (a,b) adapted from Tomasello *et al*.^16^ and Garagnani *et al*.^18^). (**c**) Micro-connectivity structure of one of the 7,500 single excitatory neural elements modelled (labelled ‘*e*’). Within-area excitatory links (in grey) to and from cell *e* are limited to a local (19 × 19) neighbourhood of neural elements (light-grey area). Lateral inhibition between *e* and neighbouring excitatory elements is realised as follows: the underlying cell *i* inhibits *e* in proportion to the total excitatory input it receives from the 5 × 5 neighbourhood (dark-purple shaded area); using analogous connections (not depicted), *e* inhibits all of its neighbours (panel (c) adapted from Garagnani and Pulvermüller^143^).

At the cellular level, the neural network implements physiologically realistic spiking neurons, and at the system level, twelve areas of relevance for language and semantic processing situated in the frontal, the temporal and the occipital lobes (see Fig. 2a). The implemented area-intrinsic, as well as between-area, connectivity was guided by prior neuroscience evidence^46,47^. Six of the areas were in the left perisylvian cortex [superior temporal Brodmann areas (BAs) 41, 42, 22 and inferior frontal areas, BAs 44, 45/6, 4], which is known to be most crucial for spoken language processing^6,15,48,49^.

- The model’s ‘auditory superior temporal stream’ included the primary auditory cortex (A1), auditory belt (AB), and modality-general parabelt areas (PB), and
- its ‘articulatory inferior frontal stream’ comprised the inferior part of primary motor cortex (M1_i_) inferior premotor (PM_i_) and multimodal prefrontal motor cortex (PF_i_).

Six additional extrasylvian areas were used to model referential meaning-related information about visual object identity (occipital-temporal ‘what’ visual stream, BAs 17, 18, 20, 21)^50^, and about executable manual actions (lateral/superior frontal areas, BAs 4, 6, 8)^51–54^.

- The ‘ventral visual stream’ included the primary visual cortex (V1), temporo-occipital (TO) and anterior-temporal areas (AT) and
- the ‘dorsolateral motor stream’, corresponding lateral primary motor (M1_L_) premotor (PM_L_), and prefrontal cortices (PF_L_).

For clarity, we will mark area labels by an asterisk when speaking about model areas (e.g. *V1), whereas the conventional labels are used for the areas in the cortex (V1). Single-neuron properties, synaptic plasticity rule, and single-area model structure are specified in more detail in the Methods section under ‘*Structure and function of the spiking neuron model’* and in previous publications^16,55^.

Briefly, the following biological, anatomical and physiological features of the cerebral cortex were replicated in the model:

(i) Neurophysiological dynamics of spiking pyramidal cells including temporal summation of inputs, threshold-based spiking, and adaptation^56,57^;
(ii) Synaptic modification by way of Hebbian-type learning, including both long-term potentiation and depression (LTP, LTD)^58^;
(iii) Local lateral inhibition and area-specific regulation mechanisms (called ‘local and global control’ below)^59,60^;
(iv) Within-area connectivity: a sparse, random and initially weak connectivity was implemented locally, along with a neighbourhood bias towards close-by links^61,62^;
(v) Between-area connectivity based on neurophysiological principles and motivated by neuroanatomical evidence^46,47^ further explained below; and
(vi) Presence of ongoing uniform uncorrelated white noise in all neurons during all phases of learning and retrieval^63^, and additional static noise added to the stimulus patterns to mimic realistic variability of input conditions during learning and retrieval.

The network’s connectivity structure reflects existing anatomical pathways revealed by neuroanatomical studies using diffusion tensor and diffusion-weighted imaging (DTI/DWI)^46,47^. These were modelled between adjacent cortical areas within each of the 4 ‘streams’ (see black arrows Fig. 2a,b) and between all pairs of multimodal areas (PB, PF_i_, AT and PF_L_) through the long distance cortico-cortical connections (purple arrows). Additionally, non-adjacent second-order ‘jumping’ links were implemented within the superior and inferior temporal and superior and inferior frontal cortices (blue arrows). Detailed descriptions of the connectivity structure and the neuroanatomical evidence reporting such links are documented in the Methods section under ‘*The model’s connectivity structure’*.

### Word learning results

**Figure 3:**
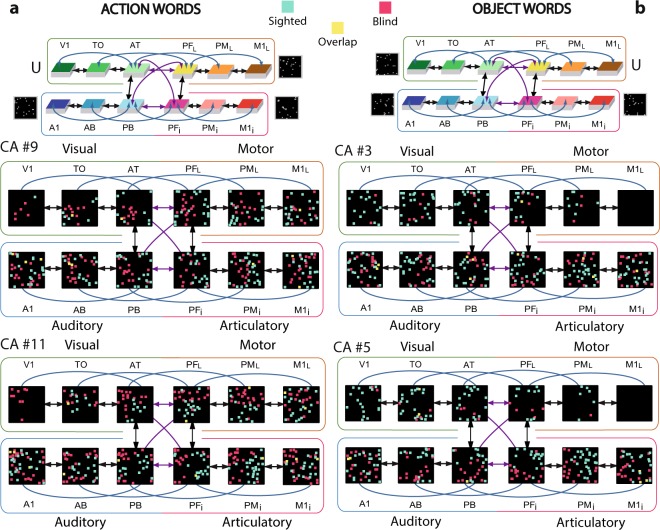
Distributions of cell assembly (CA) circuits after word learning of the blind and sighted model. CAs of action-related (**a**) and object-related (**b**) words acquired under normal (sighted, turquoise pixel) and deprived (magenta pixel) conditions. Each set of 12 squares (in black) illustrates one specific network area, with coloured pixels indexing the distribution of CA neurons across the 12 network areas as a result of sensorimotor pattern presentations. The perisylvian cortex was always stimulated, which mimics the learning of a spoken word form characterised by articulatory-acoustic features, while action words received concordant stimulation to the motor area (*M1_i_), object words were grounded to visual areas (*V1). The symbol ‘U’ indicates the uncorrelated pattern presentation simulating variable sensory or motor input typically occurring during word learning (see Methods section for more detail). The blind model was trained in the same way, but without any visual input during the entire learning phase.

Thirteen different instances of ‘sighted’ and ‘blind’ model networks (in total 26 networks) were initialised having the same architecture as described above (Fig. 2b), but each with randomly generated synaptic connections and stimulation patterns. These model instances were used to simulate plastic changes in normal-sighted and congenitally blind humans during early stages of word learning. We mimic associative learning between word forms used to speak about objects and their referent objects present in the environment as well as between action words and the performance of their semantically-related actions, as it is well-documented in the literature on language learning^37,64^. Although other forms of semantic learning (e.g., from texts or by definition) also play a role in meaning acquisition, we focus on the direct semantic grounding of words in object and action knowledge, because it is both prominent in early language learning and a precondition for other forms of semantic learning^65,66^. In the sighted model simulations, object- and action-related word acquisition was grounded in sensorimotor information presented to the primary areas of the model: object-related word learning was driven by perisylvian activity in *A1 and *M1_i_ and concordant visual (*V1) activity patterns; similarly, action-related word learning was driven by semantic activity in the lateral motor area (*M1_L_) along with perisylvian activity (Fig. 3). The fourth non-relevant area (*M1_L_ for object- and *V1 for action-related words) received an uncorrelated input pattern that differed in each  learning episode. This aimed to mimic variable input patterns uncorrelated with the word form, reflecting, for example, the many different objects that can be grasped - and visually perceived - during the acquisition of the meaning of ‘grasp’, or the different motor outputs that might occur during the learning of novel concrete (object) words unrelated to actions. In contrast, the congenitally blind models were trained with the same parameters but without any visual input during the entire learning processes (i.e., no correlated *or* uncorrelated input to *V1).

Learning the association of word forms in perisylvian language areas with the related referential semantic information in the extrasylvian system in sighted and congenitally blind models led to the formation of ensembles of strongly interconnected neurons, the so-called ‘cell assemblies’ (CA) once envisaged by Hebb (1949)^67^. These were scattered across several areas of the multi-area networks. After the learning had been completed, the CA neurons were identified by simulating ‘word production’ processes by presenting the auditory-articulatory word form patterns in the primary perisylvian areas (see Method section ‘*Data processing and statistical analysis’* for more details). Figure 3 illustrates distributions for CAs underpinning 2 object- and 2 action-related words learned under undeprived (turquoise pixels) and deprived conditions (magenta pixels; other simulated networks led to similar topographies).

**Figure 4:**
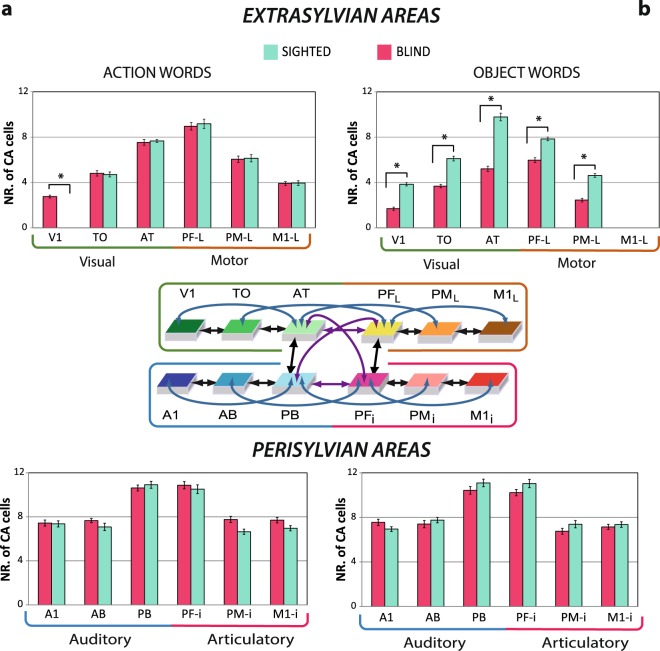
Mean numbers of cell assembly neurons in the different cortical areas. Sighted (turquoise bars) and blind (magenta bars) models after simulating the learning of action- (**a**) and object-related words (**b**); error bars show standard errors across networks. Bar graphs plot data from the extrasylvian (top) and perisylvian (bottom) systems. Asterisks indicate that, within a given area, the number of CA cells significantly differed between the sigthed and blind model for the two word types (Bonferroni-corrected planned comparison tests).

Visual inspection of the results suggested that the two types of word-related circuits did not differ in distribution across the perisylvian part of the networks. Likewise, sighted and blind model architectures produced similar perisylvian CA topographies (Fig. 3). This observation was confirmed by counts of CA neurons per area (see bar plots in Fig. 4) and by statistical results failing to support a difference in perisylvian CA distributions between word or network types. In contrast, the extrasylvian regions of the sighted model revealed a clear double dissociation between the two word types. CAs carrying object-related words seemed to extend more into the visual areas (*V1, *TO) and less into the motor areas (*PM_L_, *M1_L_), whereas action-related words showed the opposite pattern. Intriguingly, the CA circuits for action-related symbols in the blind model not only reached into the motor cortices (*PM_L_, *M1_L_) - to a similar degree as in the sighted model -, but also extended into the visual areas, including higher order and primary visual regions (*TO, *V1). The blind model’s object-word CA circuits also reached the visual system, although no (correlated or uncorrelated) visual input pattern had been presented during learning.

The bar plots in Fig. 4 show the number of CA neurons of action- (a) and object-words (b) circuits situated in extrasylvian and perisylvian systems for sighted (turquoise) and blind (magenta) models. Visual illustration comparisons of the word-related CA circuit distributions between sighted and blind models in the extrasylvian system (see bar plots in Fig. 4) show a higher CA circuit densities in the primary visual area (*V1) for action-related words in the deprived condition, which is consistent with the range of studies mentioned in the introduction about language processing in congenitally blind people. In contrast, object-related words seem to differ in all the areas of the extrasylvian system, i.e., they reveal a relatively lower neuron densities of CAs in the deprived condition.

**Figure 5:**
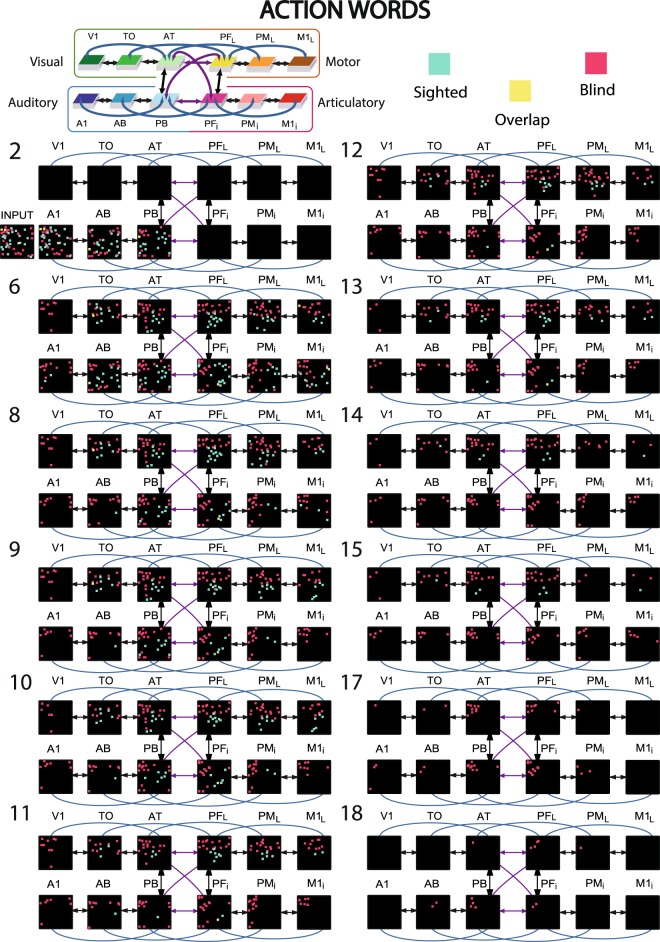
Activation spreading in the 12 area networks during simulated action word recognition. Network responses to stimulation of *A1 with the ‘auditory’ patterns of the learned words (CA #11 in Fig. 3, respectively); the 12 network areas are represented as 12 squares, but, in this case, selected snapshots of network activity are shown (as in Fig. 3) with numbers indicating the simulation time-steps. Each pixel represents one spike of the CA circuit for sighted (turquoise pixel) and blind networks (magenta pixels). Notice the prolonged spiking activation of the blind model compared to the sighted one. See main text for details.

Figure 5 illustrates the correlates of action word recognition in sighted and blind models after training. The re-activation was simulated by presenting the auditory patterns of previously learned word forms to the primary auditory area (*A1, Fig. 5). Similar to the CA structure illustrated in Fig. 3, action-related words in the blind model induced a higher number of active CA cells in the deprived visual areas compared to the sighted one. Intriguingly, the blind model revealed a prolonged activation time course (CA ignition) compared to the sighted model. In this particular example, the different neuronal and cognitive correlates of word *perception* (stimulation), word *understanding* (full ignition) and verbal working *memory* (reverberation) lasted more than 25 percent longer in the blind model as compared to the sighted one.

The observations described above were confirmed by a 3-way repeated measurement ANOVA with the factors Model (sighted/blind), WordType (action/object) and Area (6 level: primary, secondary and central areas), which revealed a main effect of Model (F_2,24_ = 11.91, *p* = 0.0047, *η*_p_^2^ = 0.49) and a significant interaction between all three factors (F_2,24_ = 13.32, ε = 0.43, *p* < 0.00001, *η*_p_^2^ = 0.52). Consistent results were revealed by the 5-way ANOVA breaking down the areas into cortical streams, which showed a significant 5-way interaction between Model, WordType, PeriExtra, TemporalFrontal and Area (F_2,24_ = 7.45, ε = 0.83 *p* = 0.0054, *η*_p_^2^ = 0.38). To further investigate this complex effect, the interaction was broken down into component analyses (4- and 3-way ANOVAs), as specified below.

First, we performed separate ANOVAs on the peri- and extrasylvian systems. A significant interaction was found in the extrasylvian system involving the factors Model, WordType, TemporalFrontal and Area (F_2,24_ = 21.46, ε = 0.82, *p* < 0.0001, *η*_p_^2^ = 0.65), while, as expected, no significant differences were revealed in the perisylvian system (F_2,24_ = 0.389, *p* = 0.68). 3-way ANOVAs investigating performance on the two word categories separately showed significant interactions of the factors Model, TemporalFrontal and Area for both action (F_2,24_ = 21.46, ε = 0.73, *p* < 0.0001, *η*_p_^2^ = 0.64) and object (F_2,24_ = 14.99, ε = 0.80, *p* < 0.0001, *η*_p_^2^ = 0.55) words. The Bonferroni-corrected planned comparison tests (6 comparisons, corrected critical *p* < 0.0083) confirmed the observation of the highest neuron density of action-related CA circuits in the blind compared to the sighted model in the primary visual area (*V1, *p* < 0.0001), whereas, for object-related word CAs, a relatively lower neuron density was revealed in the primary visual (*V1), temporo occipital (*TO), anterior temporo (*AT), lateral prefrontal (*PF_L_) and lateral premotor (*PM_L_, *p* < 0.0001) areas (Fig. 4).

To contrast the different distributions of CA neurons across areas within each model separately, we ran another set of 4-way ANOVAs with the two level factors WordType, PeriExtra, TemporalFrontal and for the factor Area (now 3 level) for the blind and for the sighted models. The sighted model showed a significant interaction between WordType and Area (F_2,24_ = 19.07, ε = 0.41, *p* < 0.001, *η*_p_^2^ = 0.72) and a significant interaction involving all four factors (F_2,24_ = 19.07, ε = 0.41, *p* < 0.001, *η*_p_^2^ = 0.62), which confirms differences in CA distributions between the two word types. Additionally, a main effect of Area (F_2,22_ = 747.838, ε = 0.98, *p* < 0.0001, *η*_p_^2^ = 0.98) was found, indicating the different CA cell densities distributed across the multi-area network, namely higher CA densities in hubs than in secondary areas (*p* < 0.0001), and in secondary than in primary areas (*p* < 0.0001). To determine whether differential CA distributions were present in peri- or extrasylvian systems, we separately ran further 3-way ANOVAs. The extrasylvian system showed a highly significant interaction of the factors WordType, TemporalFrontal and Area (F_2,24_ = 78.3, ε = 0.91, *p* < 0.0001, *η*_p_^2^ = 0.86), confirming the distinct word category distribution over the motor, visual and hub areas. The perisylvian regions did not show any significant distributional differences between the two word types (F_2,24_ = 0.46, *p* = 0.63).

The blind model showed a 2-way interaction involving WordType and Area (F_2,24_ = 19.07, ε = 0.43, *p* < 0.001, *η*_p_^2^ = 0.63), but the 4-way interaction of the factors WordType, PeriExtra, TemporalFrontal and Area was only marginally significant (F_2,22_ = 3.47, ε = 0.95, *p* = 0.054). The additional statistical analysis performed separately on the two systems showed similar results as in the sighted model, supporting distributional differences of CA topographies in extrasylvian (F_2,24_ = 13.0, ε = 0.88, *p* = 0.0003, *η*_p_^2^ = 0.51) but not perisylvian (F_2,24_ = 0.14, *p* = 0.86) space. Bonferroni-corrected planned comparison tests assessed the presence of distributional differences between word types in the blind model area by area (6 comparisons, corrected critical *p* < 0.0083). This analysis revealed higher neuron densities for action- compared to object-related words in the dorsal motor stream, i.e. in lateral prefrontal (*PF_L_, *p* < 0.0001), premotor (*PM_L_, *p* < 0.0001) and primary motor cortex (*M1_L_, *p* < 0.0001), and, surprisingly, also in the ventral visual stream, anterior-temporal (*AT, *p* < 0.0001), temporo-occipital (*TO, *p* = 0.0027) and primary visual (*V1, *p* = 0.0048) areas.

In summary, our neurobiologically constrained model of human cortex applied to simulate aspects of early word learning in congenitally blind and undeprived human individuals revealed the following results: Whereas in the undeprived case, contingencies between word forms and actions or perceptions were mapped in the network by establishing tightly interconnected neuronal assemblies distributed across linguistic, ventral visual and dorsal motor streams, comparable semantic mapping was only possible for action-related symbols in the blind model. Compared with the circuits for action-related words in the undeprived case, ‘blind networks’ showed an unexpected extension of these circuits into visual areas, with significantly higher neuron densities in primary (*V1)visual cortex. Circuits of object-related words showed relatively reduced neuron densities in both extrasylvian streams.

## Discussion

Activation of ventral stream visual cortex has been reported in healthy sighted subjects for the processing of object- and visually-related words specifically^8,34,35^, but not or significantly less in action verb and tool word processing. In contrast, congenitally blind people were shown to activate visual areas, including the primary visual cortex, in semantic retrieval during verb generation^22–26^, single word comprehension^27,28^ and sentence processing tasks^29,30^. Involvement of visual cortices in the healthy brain can be explained by their role in grounding symbolic meaning in visual perception of objects and their features^6,68,69^. However, under sensory deprivation, it is impossible that the correlation between visual and linguistic information leads to the strengthening of neuronal links into visual streams because blind people lack such modality-specific grounding information.

Here, we show that a spiking neural network constrained by cortical neuroanatomy and function and obeying well-established neuroscience principles can simulate the known visual cortex recruitment in both sighted and blind individuals during word meaning acquisition. The neuromechanistic explanatory account that we wish to offer based on these network simulations builds upon two mechanisms.

### First, CA circuits grow spontaneously

In a network with random connectivity between spontaneously active neurons, a neuron firing above the level of its connected neighbours will strengthen its links to some of these neighbours, therefore giving rise to the spontaneous emergence of a relatively more strongly connected set of neurons^70^. We call this process, which is explained by correlation learning between co-active neurons, ‘Doursat-Bienenstock expansion’ or DB-expansion. If such expansion happens at the level of large neuronal assemblies, these circuits will ‘grow into’ adjacent and connected areas^16–18^.

### Second, noise suppresses spontaneous CA circuit growth

Stimulus- and action-induced uncorrelated activity in the extrasylvian streams of the network is critical for preventing the expansion of CA circuits into these streams. In this sense, it is the variability of visual inputs in processing action-related symbols that guarantees variable activation in the visual stream and therefore neural activity uncorrelated to these symbolic-linguistic activations. For instance, when learning the meaning of an action word such as ‘run’ while performing the corresponding action^64^, the sensory information perceived during running can be seen as variable uncorrelated input, which works against DB expansion into the ventral visual stream.

Our present simulations suggest that it is the *absence of uncorrelated input to the ventral visual stream in the blind network* and brain that is necessary for DB-expansion of action-word-related CA circuits. In essence, as observed in previous simulations^16–18^, the uncorrelated visual input is crucial for preventing DB-expansion of action-word-related circuits into visual areas of the undeprived brain.

We propose that the strong activation of primary visual areas in language processing observed in congenitally blind people is explained by the DB-expansion of CA circuits described above. The relatively weaker visual activation in language processing in healthy people is explained by noise-related CA growth suppression. As mentioned in the Introduction, neuroimaging studies documented relatively stronger activation of the primary visual area (fMRI activity in V1) in blind than in undeprived individuals when generating semantically related verbs to given nouns^22–24^. Consistently, a study employing transcranial magnetic stimulation (TMS) in the primary visual area reported impairments in the verb generation task in blind but not in sighted individuals^25^. The verb generation task implies the activation of multiple CA circuits for verbs, most of which are action-related^71^, and this engages the ventral visual system more in blind people than in undeprived control subjects. Stronger V1 activation in blind than in sighted people has also been reported during sentence processing (see Fig. 1), which likely included action-related words too^29,30^. Therefore, the aforementioned fMRI and TMS results are consistent with the predictions of the present simulations, in which the modelled primary visual area (*V1) becomes more actively involved in the processing of action-related meaningful symbols and complex utterances including such symbols (Figs 1 and 3). These results represent a significant advance in the debate about the mechanisms underlying the neural changes in the visual cortex: evidence indicates that such cortical areas can take over a particular function depending on input information received during the developmental period^45^; On the basis of our results, it is precisely the lack of informative input to visual cortex that drives the Hebbian synaptic modifications and consequent extension of linguistic representations into visual cortex seen in congenitally blind individuals. The underlying mechanisms are consistent with general neurobiological plasticity principles documented in other deprived sensory systems^72,73^ and, even though a higher cognitive function, language, is involved, the explanation rests on the same neuroscience principles.

Intriguingly, the present neurobiologically constrained ‘blind’ neural network was not only able to reproduce the visual cortex recruitment in the blind but also showed prolonged spiking neural activity for action-related words during word recognition simulations (Fig. 5). Sustained neural activity is a neural correlate of working memory^74,75^, which, in the present study, persisted longer in the blind compared to the sighted model. This phenomenon in the network is consistent with the observation of enhanced verbal working memory performance in congenitally blind individuals compared to control sighted ones^22,31–33^. Note, furthermore, that during the reverberation phase, activity retreats from modality-specific to the modality general association cortices in frontal and temporal cortex (*AT, *PF) in both sighted (time steps 12–14) and blind models (time steps 17–19). This is consistent with, and provides an explanation for, the so-called ‘anterior shift’ of cortical activation from sensorimotor cortices to temporal and prefrontal connector hub regions during working memory^16,74,76,77^.

In the present simulation of undeprived referential-semantic learning, CA circuits emerged spontaneously across the fronto-temporo-occipital areas of the spiking neural network linking word-form in the perisylvian cortex with semantic information about referent objects and actions in the extrasylvian system. The learning of object- and action-related words was grounded in correlated sensorimotor information presented in the primary cortices of the architecture: besides perisylvian *A1 and *M1_i_ activity, object-related words received concordant visual (*V1) and, similarly, action-related words received lateral motor area (*M1_L_) grounding activity. Because of noise suppression of CA growth, the fourth ‘non-relevant’ input area (*M1_L_ for object- and *V1 for action-related words) was not left void of any sensory input, but instead processed uncorrelated (‘suppressing’) information and neuronal activation patterns. As reported by the present and previous simulations, noise-suppression of CA growth becomes relevant in the undeprived brain’s formation of category-specificity of circuit topographies with action-related word circuits reaching into the motor cortices (*M1_L_-*PM_L_), but not or less into visual areas (*V1, *TO), and vice versa for object words^16–18^. Here we replicated these previous results with a spiking neural network and went one step further by systematically investigating the consequences of *not* presenting such uncorrelated noise patterns to the model’s primary visual cortex during action-word learning. This was meant to specifically simulate a learning situation in which the meaning of such action words is acquired in the absence of any visual input (i.e., in blindness).

The current observations and their possible explanation in terms of DB-expansion of CA circuits and noise-related suppression of such growth suggest that these mechanisms are more broadly applicable to cases of sensory deprivation. Similar to blind individuals, deaf individuals activate their deprived auditory cortex in processing visual stimuli^78^ and in the processing of visually presented units of their native language, typically a manual signing system^79^. Some of these results had previously been used to strongly argue for an inborn mechanism linking abstract (but not acoustic or other sensory or motor) features of language to specific brain parts. Our present work offers an alternative explanation based on established neurobiological mechanisms (see Results, points (i) – (v) – (vi)).

For object-related words, simulation results indicate a generally reduced relevance of extrasylvian areas in blind people – both compared with action words in the same population and compared with the same word type in the healthy undeprived (see Fig. 4). This suggests reduced grounded semantic knowledge in blind people, at least for some specific word types requiring visual knowledge for complete acquisition of their related concepts. For the semantics of colour terms, such partially deficient semantic knowledge in the blind has been supported by experimental studies^80,81^, although other work reported comparable semantic similarity ratings^82^. However, for other object-related words, it is less plausible that substantial differences in semantic knowledge are present between congenitally blind and sighted infants. It is known that, when blind people learn words for objects, they naturally draw more on manual exploration and touch than undeprived individuals. In her seminal studies, Gleitman noted, for example, that, when a blindfolded undeprived child is advised to ‘look up’, it would raise its head, whereas a blind one would explore the space above its head with the hands^83^. This and similar observations suggest that, for a range of words typically grounded in visual experience, congenitally blind individuals use tactile and motor knowledge in the semantic grounding process. This difference in stimulation modality implies a degree of similarity between semantic grounding processes of object and action words in the blind. On the other hand, this difference in modality also implies that congenitally blind people can use similar grounding information for object words as healthy subjects, although this same (or very similar) information is provided through a different channel. This is particularly the case if information about the form or shape of referent objects is acquired through vision or tactile exploration. Future experimental works and simulation studies are still needed to explore more closely the learning of different subtypes of visually-related words in blind brains and networks taking into account, in particular, information in the tactile modality. Instead of aiming at capturing such fine-grained differences in semantic grounding, our present study specifically addressed the effect of sensory deprivation and the consequent conquering of visual cortex by linguistic and semantic processes.

We wish to conclude by pointing to further obvious limitations of the present work. First, we simulated semantic learning in a ‘grounding’ context, where words are co-present with actions and objects. Useful next steps in the modelling effort shall focus on the acquisition of novel word meaning in the context of already grounded meaningful words^84,85^ and on the learning of word sequences and whole constructions along with their semantics. With regard to blind individuals, we have restricted our scope to congenitally blind subjects, because they provide the clearest case of deprivation. The more complex situation of later deprivation, where normal learning takes place first and deprivation kicks in at a later stage, may also provide a basis for fruitful future simulations. We note that there are some important differences in reorganisation processes between congenitally, early and late blind persons^23,86,87^, which may be attributed to altered learning histories and possibly also to altered neural substrates and plasticity at different developmental stages. In spite of its focus on only one type of semantic learning and only the most typical type of visual sensory deprivation, our model offers a novel neurobiological explanation of the linguistic recruitment of visual cortex.

In sum, the present study aimed to simulate the effect of visual deprivation on the neuronal mechanisms of semantic and language processing in sighted and congenitally blind people by means of a neurobiological constrained neural network of the frontal, temporal and occipital lobes. Specifically, we focus on the mechanisms responsible for the activation of the deprived areas during semantic processing consistently reported by a number of experimental studies described above, and show that the interaction of three main factors may lead to the takeover of visual cortex for linguistic and semantic processing: (i) the changes in the balance of activity related to the absence of uncorrelated sensory input, (ii) constrained neuroanatomical connectivity and (iii) Hebbian correlation learning. Mechanisms of DB-expansion (resulting from (ii–iii)) are crucial for visual cortex recruitment in the blind, and those of ‘noise’-related prevention of such expansion for the category-specific nature of semantic circuits in healthy individuals. The present architecture explains action-related word processing in both dorsal motor and deprived ventral visual streams. Here we bridge the gap between neural mechanisms and conceptual brain functions, offering a biological account of visual cortex reorganization following sensory loss from birth and its functional recruitment for language and semantic processing.

## Methods

### Structure and function of the spiking neuron model

Each of the 12 simulated areas is implemented as two layers of artificial neuron-like elements (‘cells’), 625 excitatory and 625 inhibitory, thus resulting in 15,000 cells in total (see Fig. 2b,c). Each excitatory cell ‘*e’* consists of a leaky integrate-and-fire neuron with adaptation and simulates a single pyramidal cell representative of excitatory spiking activity in a cortical micro-column, while its twin inhibitory cell ‘*i’* is a graded-response cell simulating the average inhibitory response of the cluster of interneurons situated in a local neighbourhood^88,89^. The state of each cell *x* is uniquely defined by its membrane potential *V*(*x*, *t*), specified by the following equation:

1 $$\tau \cdot \frac{dV(x,t)}{dt}=-V(x,t)+{k}_{1}({V}_{In}(x,t)+{k}_{2}\eta (x,t))$$

where *V*_*In*_ (*x*, *t*) is the net input acting upon cell *x* at time *t* (sum of all inhibitory and excitatory postsynaptic potentials – I/EPSPs; inhibitory synapses are given a negative sign), *τ* is the membrane’s time constant, *k*_1_, *k*_2_ are scaling values (see below for the specific parameter values used in the simulations) and *η*(*·*,*t*) is a white noise process with uniform distribution over [−0.5, 0.5]. Note that noise is an inherent property of each model cell, intended to mimic the spontaneous activity (baseline firing) of real neurons. Therefore, noise was constantly present in all areas, in equal amounts (inhibitory cells have *k*_2_ = 0, i.e., the noise is generated by the excitatory cells in the model for convenience).

The output (or transformation function) ϕ of an excitatory cell e is defined as follows:

2 $$\varphi (e,t)=\{\begin{array}{cc}1 & if\,(V(e,t)-\alpha \,\omega (e,t)) > thresh\\ 0 & otherwise\end{array}$$

Thus, an excitatory cell *e* spikes (=1) whenever its membrane potential *V*(*e*, *t*) overcomes a fixed threshold *thresh* by the quantity *αω*(*e*, *t*) (where *α* is a constant and *ω* is defined below). Inhibitory cells are graded response neurons as they intend to represent the average impact of a cluster of local interneurons; the output *ϕ*(*i*, *t*) of an inhibitory neuron *i* is 0 if *V*(*i*, *t*) <0 and *V*(*i*, *t*) otherwise.

To simulate neuronal adaptation^90^, function *ω*(*·*,*t*) is defined so as to track the cell’s most recent firing rate activity. More precisely, the amount of adaptation *ω*(*e*, *t*) of cell *e* at time *t* is defined by:

3.1 $${\tau }_{ADAPT}\cdot \frac{d\omega (e,t)}{dt}=-\omega (e,t)+\varphi (e,t)$$

where ${\tau }_{ADAPT}$ is the ‘adaptation’ time constant. The solution *ω*(*e*, *t*) of Eq. (3.1) is the low-pass-filtered output *ϕ* of cell *e*, which provides an estimate of the cell’s most recent firing-rate history. A cell’s average firing activity is also used to specify the network’s Hebbian plasticity rule (see Eq. (4) below); in this context, the (estimated) instantaneous mean firing rate *ω*_*E*_(*e*, *t*) of an excitatory neuron *e* is defined as:

3.2 $${\tau }_{Favg}\cdot \frac{d{\omega }_{E}(e,t)}{dt}=-{\omega }_{E}(e,t)+\varphi (e,t)$$

Local (lateral) inhibitory connections (see Fig. 2c) and area-specific inhibition are also implemented, realising, respectively, local and global competition mechanisms^91,92^. More precisely, in Eq. (1) the input *V*_*In*_(*x*, *t*) to each excitatory cell of the same area includes an area-specific (‘global’) inhibition term *k*_*G*._*ω*_*G*_(*e*, *t*) (with *k*_*G*_ a constant and *ω*_*G*_(*e*, *t*) defined below) subtracted from the total I/EPSPs postsynaptic potentials *V*_*In*_ in the input; this regulatory mechanism ensures that area (and network) activity is maintained within physiological levels^59^:

3.3 $${\tau }_{GLOB}\cdot \frac{d{\omega }_{G}(e,t)}{dt}=-{\omega }_{G}(e,t)+\sum _{e\in area}\varphi (e,t)$$

Excitatory links within and between (possibly non-adjacent) model areas are established at random and limited to a local (topographic) neighbourhood; weights are initialised at random, in the range [0, 0.1]. The probability of a synapse to be created between any two cells falls off with their distance^59^ according to a Gaussian function clipped to 0 outside the chosen neighbourhood (a square of size *n* = 19 for excitatory and *n* = 5 for inhibitory cell projections). This produces a sparse, patchy and topographic connectivity, as typically found in the mammalian cortex^59,61,93,94^.

The Hebbian learning mechanism implemented simulates well-documented synaptic plasticity phenomena of long-term potentiation (LTP) and depression (LTD), as described by Artola, Bröcher and Singer^58,95^. This rule provides a realistic approximation of known experience-dependent neuronal plasticity and learning^96–98^, and includes both (homo- and hetero-synaptic, or associative) LTP, as well as homo- and hetero-synaptic LTD. In the model, we discretized the continuous range of possible synaptic efficacy changes into two possible levels, +Δ and −Δ (with Δ≪1 and fixed). Following Artola *et al*., we defined as ‘active’ any (axonal) projection of excitatory cell *e* such that the estimated firing rate *ω*_*E*_(*e*, *t*) of cell *e* at time *t* (see Eq. (3.2)) is above *θ*_*pre*_, where *θ*_*pre*_ ∈ [0, 1] is an arbitrary threshold representing the minimum level of presynaptic activity required for LTP to occur. Thus, given a pre-synaptic cell *i* making contact onto a post-synaptic cell *j*, the change Δ*w*(*i*, *j*) in efficacy of the (excitatory-to-excitatory) link from *i* to *j* is defined as follows:

4 $${\rm{\Delta }}w(i,j)\{\begin{array}{ccc}+{\rm{\Delta }} & if\,{\omega }_{E}(i,t)\ge {\theta }_{pre}\,and\,V(j,t)\ge {\theta }_{+} & (LTP)\\ -{\rm{\Delta }} & if\,{\omega }_{E}(i,t)\ge {\theta }_{pre}\,and\,{\theta }_{-}\le V(j,t) < {\theta }_{+} & (homosynaptic\,LTD)\\ -{\rm{\Delta }} & if\,{\omega }_{E}(i,t) < {\theta }_{pre}\,and\,V(j,t)\ge {\theta }_{+} & (heterosynaptic\,LTD)\\ 0 & otherwise & \end{array}$$

### Parameter values used during simulations

**Table:** 

Eq.(1)	Time constant (excitatory cells)	*τ* = 2.5 (simulation time-steps)
	Time constant (inhibitory cells)	*τ* = 5 (simulation time-steps)
	Total input rescaling factor	*k*_1_ = 0.01
	Noise amplitude	*k*_2_ = 1∙√(24/Δt)
	Global inhibition strength	*k*_*G*_ = 0.60
Eq. (2)	Spiking threshold	*thresh* = 0.18
	Adaptation strength	α = 7.0
Eq.(3.1)	Adaptation time constant	*τ*_*ADAPT*_ = 10 (time steps)
Eq.(3.2)	Rate-estimate time constant	*τ*_*Favg* = _30 (time steps)
Eq.(3.3)	Global inhibition time constant	*τ*_*GLOB*_ = 12 (time steps)
Eq.(4)	Postsynaptic membrane potential thresholds:	
		*θ*_+_ = 0.15
		*θ*_*–*_ = 0.14
	Presynaptic output activity required for LTP:	
		*θ*_*pre*_ = 0.05
	Learning rate	Δ = 0.0008

### The model’s connectivity structure

The between-area connectivity binds adjacent cortical areas together^99–101^. In the perisylvian system, next-neighbour connections between cortically adjacent areas are implemented within the auditory (A1, AB, PB)^102–104^, as well as within the articulatory (PF_i_, PM_i_, M1_i_) sub-systems^99,100^. Similarly, local next neighbour links are also realised in the extrasylvian system, between adjacent ventral visual (V1, TO, AT)^105,106^, and dorsolateral motor areas (PF_L_, PM_L_, M1_L_)^52–54,99,107,108^. Furthermore, reciprocal links also exist between anterior-temporal (AT) and parabelt (PB) areas^109^ and inferior and lateral prefrontal (PF_i_, PF_L_) areas^110^.

The long distance cortico-cortical connections implemented reciprocally link all pairs of multimodal hub areas (PB, PF_i_, AT and PF_L_) of the four sub-systems, modelling documented anatomical connections between inferior pre-frontal (PF_i_) and auditory parabelt (PB)^111–117^ and between anterior-temporal (AT) and lateral prefrontal (PF_L_) areas, realised by the arcuate and the uncinated fascicles^118–124^. The peri- and extrasylvian systems are also linked by means of long distance cortico-cortical connections across the central hub areas; likewise parabelt (PB) and lateral prefrontal cortex (PF_L_) are reciprocally connected^117,125,126^ as well as the anterior/middle-temporal (AT) and inferior prefrontal (PF_i_) areas^118,119,125,127–129^.

The present neural spiking network implemented additional second-order ‘jumping’ links, which skip one intermediate area (blue arrows Fig. 2b), documented by a range of recent neuroanatomical and diffusion tensor and diffusion-weighted imaging (DTI/DWI) studies in humans and non-human primates. These links exist within (auditory) superior temporal and (articulatory) inferior frontal cortex of the perisylvian cortex, that is amongst: primary auditory (A1) - parabelt (PB) areas^99,101^, parabelt (PB) - inferior premotor (PM_i_) areas^130^, auditory belt (AB) - inferior prefrontal (PF_i_)^102,126,131^ and as well inferior prefrontal (PF_i_) - primary motor (M1_i_) areas^100,132,133^. Additional evidence for the presence of second-order jumping links within the perisylvian system are well-documented also in DTI/DWI studies in humans^46,47^. The ventral visual and the dorsolateral motor sub-systems of the extrasylvian cortex were also endowed with jumping links, similarly to the perisylvian cortices listed above. In particular, primary visual (V1) area is reciprocally linked to anterior-temporo (AT) area^134,135^, as well as anterior-temporo (AT) and dorsolateral premotor (PM_L_) area, as documented by both anatomical^125,136^ and monkey studies^121,122,137^. Additional jumping links were implemented between temporo-occipital (TO) and dorsolateral prefrontal areas (PF_L_), as supported by evidence from anatomical studies in humans^113^ and monkeys^120,122,136,138^, and between dorsolateral prefrontal (PF_L_) and dorsolateral premotor (M1_L_) areas^100,132,133^. Further neuroanatomical DTI studies also showed connections within the extrasylvian system as described above^47^. Notice that the connectivity structure of both sighted and blind models was kept the same, as a number of DTI studies have shown similar anatomical connectivity structure between sighted and blind populations^38–41^.

### Simulating word learning

Prior to the training, each network was initialised with all the synaptic links (between- and within-areas) connecting single cells established at random (see Methods section under ‘*Structure and function of the spiking neuron model’*). Similar to previous simulation studies^16–18,55^, word-meaning acquisition was then simulated under the impact of repeated sensorimotor pattern presentations to the primary areas of the network. Each network instance used 12 different sets of sensorimotor word patterns representing six object- and six action-related words. Each pattern consisted of a fixed set of 19 cells chosen at random within the 25 × 25 cells of an area (ca. 3% of the cells). Note that additional white (so-called ‘contextual’) noise was continuously presented to all primary areas of the network, and thus superimposed on all learning patterns. This partly accounted for a degree of variability during word meaning acquisition of the two word-types.

Word-related sensorimotor patterns were presented 3000 times (previous simulations using a six area model showed no substantial change in the primary areas for between 1000 and 10000 learning steps^139,140^) as described above. A trial started with a word pattern presentation for 16 simulation time steps, followed by a period during which no input (interstimulus interval – ISI) was given. The next word pattern (learning step) was presented to the network only when the global inhibition of the PF_i_ and PB areas decreased below a specific fixed threshold; this allowed the activity to return to a baseline value, so as to minimise the possibility of one trial affecting the next one. Only the inherent baseline noise (simulating spontaneous neuronal firing) and ‘contextual’ noise were present in the neural-network during each ISI.

### Data processing and statistical analysis

Cell assemblies, which are strongly interconnected networks of neurons, spontaneously emerged during word learning simulation. After learning, the word form neurons in the primary perisylvian auditory-articulatory areas (A1, M1_i_) simulating the ‘word production’ were activated for 15 simulation time-steps to identify and quantify the neurons forming the 12 distributed CA circuits that emerged across the network areas. During this period, we computed and displayed the average firing rate of each excitatory cell (7500 *e*-cells, cell’s responses).

As an estimate of a cell’s average firing-rate here we used the value *ω*_*E*_(*e*, *t*) from Eq. (3.2), integrated with time-constant ${\tau }_{Favg}=5$. An *e*-cell was then taken to be a member of a given CA circuit only if its time-averaged rate (output value or ‘firing rate’) reached a threshold $\vartheta$ which was area- and input-pattern specific, and defined as a fraction *γ* of the maximal single-cell’s time-averaged response in that area to pattern *w*. More formally,

$$\vartheta ={\vartheta }_{A}(w)=\gamma \mathop{max}\limits_{x\in A}\bar{O{(x,t)}_{w}}$$

where $\overline{O{(x,t)}_{w}}$ is the estimated time-averaged response of cell *x* to word pattern *w* (see Eq. 3.3) in Methods section under ‘*Structure and function of the spiking model’*) and *γ* ∈ [0, 1] is a constant (we used *γ* = 0.5 on the basis of previous simulation results^17,140,141^). This was computed for each of the 13 trained network instances, averaging the number of CA cells per area over the 6 object- and 6 action-related words.

To investigate the presence of significant statistical differences between sighted and blind neural network models, we performed an initial statistical analysis including both neural network models. To this end, a 3-way ANOVA was run with factors Model (two levels: *Sighted* vs. *Blind*), WordType (two levels: *Object* vs. *Action*) and Area (12 levels: *Perisylvian* = {A1, AB, PB, M1_i_, PM_i_, PF_i_}, *Extrasylvian* cortex = {V1, TO, AT, M1_L_, PM_L_, PF_L_}). Additionally, to further investigate differences of the modelled cortical regions between the two models a 5-way ANOVA was run with factors Model (two levels: *Sighted* vs. *Blind*), WordType (two levels: *Object* vs. *Action*), PeriExtra (two levels: *Perisylvian* = {A1, AB, PB, M1_i_, PM_i_, PF_i_}, *Extrasylvian* cortex = {V1, TO, AT, M1_L_, PM_L_, PF_L_}), TemporalFrontal (TempFront) (2 levels: *Temporal* *areas* = {A1, AB, PB, V1, TO, AT}, *Frontal areas* = {M1_L_, PM_L_, PF_L_, M1_i_, PM_i_, PF_i_}) and Area (three levels: *Primary* = {A1, V1, M1_L_, M1_i_}, *Secondary* = {TO, AB, PM_L_, PM_i_} and *Central* = {PB, AT, PF_L_, PF_i_} areas). Subsequently, each system, 6 peri- and 6 extrasylvian areas, were investigated separately with factors ‘Model’, ‘WordType’, ‘TempFront’ and ‘Area’. The same statistical analysis, but this time omitting ‘WordType’ as a factor was additionally performed to disentangle the different CA distribution of action- and object-related words between the two models.

A second level of analysis was run on each Model (blind and sighted) separately, first with a 2-way ANOVA with factors ‘WordType’ and ‘Area’ and a 4-way ANOVA with factors ‘WordType’, ‘PeriExtra’, ‘TempFront’ and ‘Area’ and subsequently, with 3-way ANOVA on each system within the sighted and blind model, peri- and extrasylvian systems, separately. Corrected p-values along with epsilon (ε) values are reported throughout. Partial eta-square (*η*_p_^2^) values are also stated, which is defined as an index of effect size (0.01–0.06 small, 0.06–0.14 medium and >0.14 large^142^).

## Data Availability

The datasets generated during the current study are available in the Zenodo repository, 10.5281/zenodo.2551071.
